# The Role of Triggers in Physical Activity among College Students: An Extended Model of the Theory of Planned Behavior

**DOI:** 10.3390/bs14040328

**Published:** 2024-04-15

**Authors:** Yunbo Wang, Hyoung-Kil Kang

**Affiliations:** 1 School of Physical Education, Changchun Normal University, Changchun 130032, China; wangyunbo@ccsfu.edu.cn; 2 Department of Physical Education, Kyungnam University, Changwon 51767, Republic of Korea

**Keywords:** college students, behavioral intention, physical activity behavior, Theory of Planned Behavior, Fogg behavior model, triggers

## Abstract

Objective: This study aims to extend the Theory of Planned Behavior with Triggers (TPBT) to improve the prediction of physical activity (PA) behavior using the TPB model. Methods: Questionnaires, including the TPB scale, PA rating scale (PARS-3), and triggers scale, were administered to 596 Chinese college students, and the data were analyzed using SPSS 23.0 and AMOS 24.0. Results: Subjective norm (SN), attitude (AT), and perceived behavioral control (PBC) all significantly and positively affected behavioral intention (BI). The path coefficient of PA behavior was significantly influenced by the interaction term of three types of triggers and BI, and the TPB with Triggers (TPBT) model improved the explanation rate of PA behavior. Conclusion: Triggers have a moderating effect on the relationship between BI and PA behavior, and the TPBT model better explains college students’ PA behavior. Among the three dimensions of triggers, people are more receptive to facilitator and signal triggers than spark triggers. This has practical implications for practitioners designing interventions to promote PA among college students.

## 1. Introduction

Physical activity (PA) refers to any form of activity that uses energy generated by skeletal muscle activity [1], and regular physical activity is socially, psychologically, and physically beneficial for young people [1]. Because of the evidence-based benefits of physical activity, it has been used as an intervention strategy to improve the mental and physical health of college students [2]. Despite this, physical inactivity has become a significant public health problem affecting people worldwide [3], and the World Health Organization (WHO) considers physical inactivity to be the fourth leading risk factor for global mortality [1].

This global trend is no exception for Chinese students. Since 2010, the health level of Chinese college students has continued to decline, and the number of obese Chinese college students has steadily increased, mainly due to physical inactivity [4]. Studies have also shown that the detection rate of depression among Chinese college students is as high as 31.38%, with an increasing trend year by year [5]. This is partly due to physical inactivity, as adolescents who are physically active for more than 30 min and five or more times per week have a 60% reduction in self-reported depressive symptoms [6]. Luo and colleagues also found that Chinese college students are dangerously clinically sedentary, as prolonged sitting can cause a variety of negative effects, including increased risk of chronic diseases, reduced cardiovascular health, and decreased bone density [7]. In light of these situations, this study aims to explore ways to promote physical activity among Chinese college students by extending the Theory of Planned Behavior (TPB) with triggers.

The TPB has been widely used to analyze, explain, and predict behavior in various contexts, including education, medicine, and health [8]. The TPB comprises attitude (AT), subjective norm (SN), perceived behavioral control (PBC), and behavioral intention (BI). AT, SN, and PBC are three social cognitive characteristics that significantly influence BI. AT refers to a person’s evaluation of behavior as favorable or unfavorable, while SN refers to the perceived societal pressure that influences individuals to engage in or refrain from a particular behavior. PBC represents an individual’s perceived ability to perform a specific behavior [9]. Shen observed that AT, SN, and PBC can account for a significant amount of variation in PA intention, and each of these factors can predict PA behavior [10]. Therefore, the TPB helps us to comprehend how intrinsic psychological factors, such as AT and PBC, and extrinsic social psychological factors, reflected by SN, influence PA behavior [11].

While the TPB has been found to have a high explanatory power for BI, it has been criticized for its low explanatory power for behavior [12]. According to Conroy and colleagues, people’s BI changes over time, and the longer the time between BI and behavior, the weaker the link between the two becomes [13]. Sheeran also noted that although 47% of people have a positive BI to implement healthy behaviors, only 7% of them execute those behaviors [14]. Furthermore, Zhang and Mao reported that the percentage of teenagers engaging in PA remains low despite a high BI to engage in PA. They proposed that other factors may affect PA behavior [15]. Scholars have since explored ways to improve the explanatory power of the TPB for behavior. For instance, Feng and Mao introduced self-determined motivation into the TPB, but the improvement in explanatory power was limited [3]. Bogg and Roberts proposed that promoting desired behavior and enhancing its performance are two key considerations to strengthen the association between BI and behavior [16]. Hu found that planning can mediate the relationship between BI and behavior [17]. As such, researchers have attempted to add variables to the TPB to understand the origins of people’s behaviors better. However, the relationship between BI and behavior remains unclear and limited. Thus, this study incorporates triggers into the TPB (namely, the TPBT) to gain a better understanding of the physical activity of Chinese college students.

Triggers come from the Fogg behavior model (FBM), a behavioral design theory proposed by Fogg [18,19]. Fogg stated that the FBM allows us to understand the drivers of human behavior and proposed that human behavior can be controlled by three factors: motivation, ability, and triggers [18]. Numerous studies have examined the role of motivation and ability in human behavior, e.g., [9,20,21], and what distinguishes the FBM from previous behavioral theories [22] is the use of triggers. The triggers can be an alarm, a text message, an invitation, or someone’s encouragement for individuals to perform a target behavior. “Whatever the form, successful triggers have three characteristics: First, we notice the trigger. Second, we associate the trigger with a target behavior. Third, the trigger occurs when we are both motivated and able to perform the behavior” [18] (p. 3). Fogg conceptualized three types of triggers, including sparks, facilitators, and signals [18,19]. A spark directly stimulates the user’s demand motivation and produces self-drive to promote the occurrence of the target behavior. A facilitator provides explicit guidance for the user’s target behavior. A signal acts as a reminder or cue to remind the individual to perform the target behavior [18,23]. Fogg argues that even if individuals have both the motivation and ability to engage in physical activity, they will not change their behavior without triggers [18]. In other words, triggers are the final step in leading individuals to perform target behaviors [24] and the determining factor for the occurrence of the behavior [19].

Studies have been conducted to verify the roles of signals, sparks, and facilitators in promoting physical activity (PA). Cai [25] conducted an experimental intervention on elderly chronic disease patients with PA deficiency by providing them with smart bracelets and sending health knowledge and regular reminders through the WeChat platform. The study found a significant increase in the total amount of PA after the intervention. Li et al. [26] provided college students with daily articles and videos on physical exercise and fitness guidance through the WeChat platform. The study found that WeChat created a social and autonomous support environment, which improved the college students’ extracurricular physical activity. Similarly, Wang et al. [27] reported that peer support positively and significantly impacted adolescents’ physical activity. Li et al. [28] also demonstrated that parental, teacher, and friend support have a significant positive moderating effect on the relationship between exercise motivation and exercise persistence. Motivation to engage in physical activity and social support are found to be crucial in forming sport intention and maintaining sport behavior [29,30]. As such, various triggers have been separately experimented with and proven to be important interventions in promoting physical activity. However, studies that comprehensively address triggers are limited because a questionnaire to measure three types of triggers has only recently been developed [22]. Therefore, the purpose of this study is to examine the moderating role of three types of triggers between BI and PA behavior. The following hypotheses are proposed.

1. Three types of triggers have a moderating effect on the relationship between BI and PA behavior.
2. The TPBT model can significantly predict college students’ PA behavior and improve the interpretation rate of PA behavior.

## 2. Methods

### 2.1. Participants

This study randomly selected students of 17 physical education classes at Changchun Normal University. The research team obtained informed consent from the participants who were invited to participate in the study, explaining the purpose, process, and possible inconvenience of the research in detail. A total of 621 college students volunteered to participate in the study. After excluding incomplete questionnaires, 596 questionnaires were used for data analysis. The participants consisted of 198 males (33%) and 398 females (66%) with a mean age of 19.03 ± 0.856 years. The demographic characteristics of the participants are presented in Table 1.

### 2.2. Design and Procedure

The research team supervised and guided the administration of the questionnaires. They provided detailed instructions and distributed paper copies of the questionnaires during the participants’ PE class times. The team was available to answer any questions while the participants completed the questionnaires. After completion, the research team collected the questionnaires.

This study considered PA intention as the intention to participate in exercise within the next two weeks rather than immediate intention [31]. To measure PA intention, the participants were asked to rate their agreement with the statement ‘For the next two weeks, I plan to do at least 20 min of physical activity at least 3 times a week’. Two weeks after measuring their PA intention, the participants’ PA behavior was measured. The data were collected in two phases. During the first phase, the research team used the TPB scale and the PA behavior triggers questionnaire to measure BI, AT, SN, PBC, and the subjects’ triggers. In the second phase, which took place two weeks after the BI measurement, the participants completed the PA rating scale to evaluate their PA behavior during the previous two weeks. The research team then matched the results of the two questionnaires by student ID numbers.

### 2.3. Measures

To measure the TPB, 14 items of the Chinese version of the “Theory of Planned Behavior Scale” developed by Hu were used [17]. The scale is a 6-point Likert scale. AT was measured by 5 items (e.g., For me, 20+ min of physical activity at least 3 times a week for the next two weeks is …), with a response scale ranging from 1 (pleasant) to 6 (unpleasant). SN was measured by 3 items (e.g., Most of the people who are important to me want me to do 20+ min of physical activity at least 3 times a week), with a response scale ranging from 1 (agree) to 6 (disagree). PBC was assessed with 3 items (e.g., Do I have the ability to control myself for at least 20 min of physical activity at least 3 times a week for the next two weeks), with a response scale ranging from 1 (totally) to 6 (not at all). BI was measured by 3 items (e.g., For the next two weeks, I plan to do at least 20 min of physical activity at least 3 times a week), with a response scale ranging from 1 (strongly agree) to 6 (strongly disagree). The internal consistency reliability coefficients of the subscales of AT, SN, PBC, and BI were 0.893, 0.794, 0.858, and 0.857, respectively.

The “Physical Activity Triggers Questionnaire” compiled by Wang and Kang was used to measure triggers [22]. The questionnaire consists of 14 items on a 5-point Likert scale (1, strongly disagree; 5, strongly agree). The spark was measured by 3 items (e.g., seeing sports events or sports-related content broadcasted by public media). The facilitator was measured by 6 items (e.g., My parents invited me to do physical activities together). The signal was measured by 5 items (e.g., a timed reminder of a sports watch or mobile phone). The internal consistency reliability coefficients of spark, facilitator, and signal were 0.857, 0.906, and 0.887, respectively.

To measure PA behavior, the “Physical Activity Rating Scale (PARS-3)” developed by Liang was used [32]. The scale is a 5-point Likert scale and consists of three items, including PA intensity, PA time, and PA frequency. In this study, the amount of physical activity was measured by multiplying the intensity of physical activity by the frequency of physical activity and the duration of time minus one (the amount of PA = PA intensity × (time − 1) × PA frequency). The internal consistency reliability coefficient of the scale was 0.876.

### 2.4. Data Processing

This study used SPSS 23.0 and AMOS 24.0 for statistical analysis of the data. The paper version of the questionnaire data was first sorted and analyzed in Excel. Descriptive statistics, correlation analysis, regression analysis, and moderating effect analysis were then performed using the SPSS software. The AMOS software was used to construct a structural equation model and test the path coefficient and fit of the TPB and TPBT models.

## 3. Results

Table 2 presents the results of the Jarque–Bera normal distribution test for the study variables. According to the test, a sample is considered normally distributed if the absolute value of skewness is less than 3 and the absolute value of kurtosis is less than 10 [33,34]. The study sample exhibited a normal distribution, with skewness values ranging from 0.070 to 0.945 and kurtosis values ranging from 0.172 to 1.119.

Table 3 showed the PA behavior level of the 596 college students was 27.01 ± 26.190, which was a moderately low level. BI was significantly positively correlated with SN, AT, and PBC, including SN (*r* = 0.362, *p* < 0.01), AT (*r* = 0.491, *p* < 0.01), and PBC (*r* = 0.499, *p* < 0.01). The correlation between triggers and SN, AT, PBC, and BI was low (*r* = −0.022–0.069) and not significant. Triggers were significantly positively correlated with PA behavior (*r* = 0.262, *p* < 0.01). PA behavior and BI were strongly significantly correlated (*r* = 0.548) [35]. Correlation analysis was conducted to examine multicollinearity issues, and no multicollinearity issue was found, as all the correlation values were lower than 0.55 [36].

### 3.1. Prediction of College Students’ PA Behavior by TPB Model

**Figure 1:**
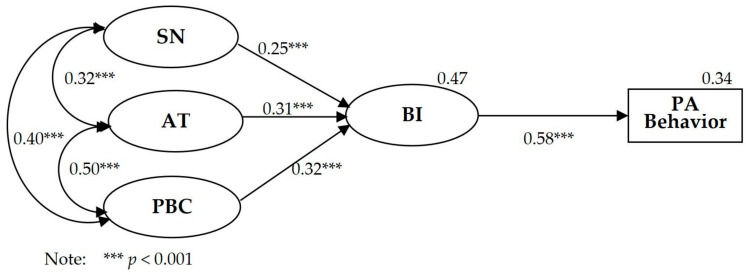
Structural equation modeling analysis of the TPB model.

Table 4 and Figure 1 show the findings of the structural equation modeling of the TPB model. Two absolute fitting indexes (*χ*^2^/*df =* 3.544 and RMSEA = 0.065) met the fitting recommendation, as the relative *χ*^2^*/df* was less than 5 and the RMSEA was less than 0.08. Three relative fitting indexes of IFI, TLI, CFI, and AGFI were greater than 0.90, all of which met the fitting recommendation [37,38]. The model fit indexes showed that the TPB model was acceptable and that the model explained BI at *R*^2^ = 0.47 and PA behavior at *R*^2^ = 0.34.

### 3.2. Prediction of PA Behavior of College Students by TPB and Triggers Model

**Figure 2:**
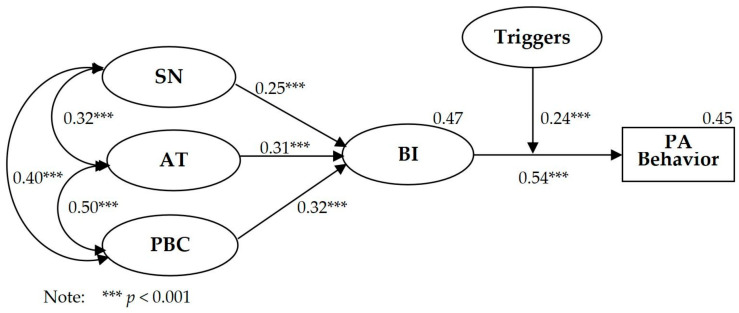
Path coefficients of TPB and triggers model.

Structural equation modeling was conducted on the TPBT model. As shown in Table 5, the two absolute fitting indicators of the TPBT model met the fitting recommendation, i.e., *χ*^2^*/df* = 2.535 (<5) and RMSEA = 0.051 (<0.08), and IFI = 0.952 (>0.90), TLI = 0.944 (>0.90), CFI = 0.951 (>0.90), and AGFI = 0.917 (>0.90) met the fitting recommendation. As shown in Figure 2, the path coefficients from SN (*β* = 0.25, *p* < 0.001), AT (*β* = 0.31, *p* < 0.001), and PBC (*β* = 0.32, *p* < 0.001) to BI were significant. The path coefficient between BI (*β* = 0.54, *p* < 0.001) and PA behavior was significant. The interaction term of triggers between BI and PA behavior (*β* = 0.24, *p* < 0.001) was significant. PBC showed the strongest predictive effect on BI, and SN showed the weakest predictive effect on BI. The TPBT model explained *R*^2^ = 0.45 for PA behavior. Compared with the TPB model, the TPBT model made an increase of *R*^2^ = 0.11 in the explanation rate of PA behavior, showing that the addition of triggers significantly increased the explanation rate for PA behavior.

### 3.3. Moderating Effect of Triggers

#### 3.3.1. Triggers Moderating Effect Test

To understand the moderating effect of triggers between BI and PA behavior in detail, the product term needed to be generated by centering the data, and then the hierarchical regression analysis was carried out [39]. After centralizing all the observed indicators of triggers and behavioral intention, whether the interaction term of triggers and BI was significant in predicting PA behavior was examined. The analysis showed that the interaction term of triggers and BI significantly affected the regression coefficient of PA behavior (*β* = 0.187, *p* < 0.001), indicating that triggers significantly moderated the relationship between BI and PA behavior (presented in Table 6).

**Figure 3:**
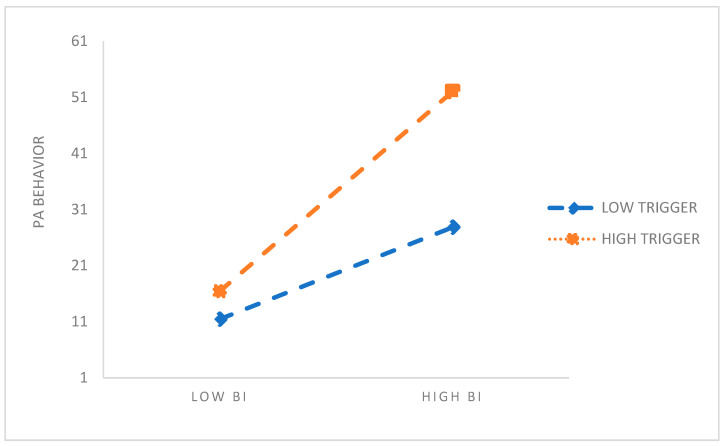
The moderating effect of triggers on BI and PA behavior.

A simple slope test was performed to plot the moderating effect according to the high and low degree of triggers (*Mean* ± 1 *SD*). As shown in Figure 3, when the trigger changed from a low value to a high value, the slope of BI on PA behavior increased, indicating that BI had a stronger positive influence on PA behavior in people with high triggers compared with those with low triggers.

#### 3.3.2. Sparks, Signals, and Facilitators Moderating Effect Test

Given that moderating effects of triggers were found, the moderating effects of sparks, signals, and facilitators were tested, respectively, (Table 7). The regression coefficient of BI × spark was significant (*β* = 0.075, *p* = 0.031 < 0.05), indicating that sparks had a significant moderating effect between BI and PA behavior, and for every 1 *SD* increase in sparks, the slope of BI to PA behavior increased by 0.075 *SD*. The regression coefficient of BI × signal was significant (*β* = 0.152, *p* < 0.001), indicating that signals had a significant moderating effect between BI and PA behavior, and for every 1 *SD* increase in signals, the slope of BI to PA behavior increased by 0.152 *SD*. The regression coefficient of BI × facilitator was significant (*β* = 0.186, *p* < 0.001), indicating that facilitators had a significant moderating effect, and for every 1 *SD* increase in facilitators, the slope of BI to PA behavior increased by 0.186 *SD*. Among the three dimensions, sparks had the weakest moderating effect compared to facilitators and signals.

## 4. Discussion

### 4.1. The Moderating Effect of Triggers

The analysis showed that the interaction term of triggers and BI significantly affected the regression coefficient of PA behavior, indicating that triggers significantly moderate the relationship between BI and PA behavior. This indicates that the three trigger dimensions (sparks, facilitators, and signals) play a significant role in the process of transforming BI into PA behavior. A trigger is something that prompts individuals to perform a behavior immediately [18]. In this study, spark items refer to sports events or sports-related content broadcasted by public media, sports advertisements, and push messages from short messages or WeChat. These spark items are associated with unexpected and indirect messages that stimulate and attract Chinese college students to engage in physical activity. The facilitator items included guidance and supervision from parents, friends, doctors, and public figures. The signals consisted of reminders set by the initiative, such as mobile phone alerts, fitness apps, alarm clocks, WeChat messages, self-posted pictures, or text messages. Specifically, this study found that sparks had the weakest moderating effect compared to facilitators and signals. This finding is consistent with Fogg’s conceptualization, which proposed that the influence of facilitators and signals on a target behavior is higher than that of sparks because sparks may push individuals to engage in behaviors that they do not want to do [18]. These results align with the TPBT model used in this study.

The finding of the moderating effect of triggers also means that the PA behavior of college students can be enhanced by triggers, which is supported by previous studies. For example, as with the signal triggers in this study, studies have shown that using sports apps and social media to intervene in PA can effectively improve PA behavior [40,41], and using WeChat to remind college students of PA was shown to improve their amount of exercise and exercise intensity [26]. As with the facilitator triggers in this study, the encouragement and support of parents, teachers, and friends is one of the main factors affecting whether teenagers can persist in carrying out physical exercise [42], and peer support has a significant positive effect on college students’ physical exercise behavior [43,44]. Li utilized female college students to create exercise support groups and discovered that social support can enhance exercise behavior [45]. As with the spark triggers in this study, publicizing the benefits of sports activities with mass media and social media has been shown to promote college students’ PA [22,46]. Quinton et al.’s [47] experiment demonstrated that sending short messages on a regular basis successfully influenced people’s attitude, intention, and behavior regarding physical activity. This study provides empirical evidence of the role of triggers in promoting physical activity behaviors in the TPBT model, and hypothesis 1 is thus supported.

According to the FBM, all behaviors can be designed. If a behavior does not occur, it should be examined from the perspectives of ability, motivation, and triggers [19]. Chinese college students are required to take a physical education exam and a national physical health test for graduation. Therefore, they have relatively higher motivation and ability for physical activity compared to college students in other countries. Thus, the key to increasing physical activity among Chinese college students may be related to triggers. Fogg suggests that motivation and ability are relatively unchangeable compared to triggers, which need to be designed based on individual needs and provided at the right time [19]. Practitioners should consider each college student’s unique trigger preferences and the optimal time to provide them when designing triggers.

The findings indicate that interventions aimed at promoting physical activity among college students should focus primarily on facilitators and signals. College students require guidance and support from parents, friends, teachers, and doctors to engage in physical activity. Parents, as the primary educators of college students, play a crucial role in cultivating their physical activity habits. Additionally, friends can provide guidance and support to promote physical activity among college students. Furthermore, students are encouraged to engage in physical activity through various apps, WeChat groups, and reminder functions. These reminders aim to motivate students who may lack the ability or motivation to engage in physical activity.

### 4.2. Integrated Model of TPB and Triggers

Previous studies have attempted to increase the explanatory power of the TPB model by adding variables such as executive function [31], planning, and self-efficacy [17,48]. However, none of these studies have incorporated the triggers of the FBM into the TPB model. This study is the first to empirically examine the TPBT model. This study demonstrates that AT, SN, and PBC explain 47% of the variance in BI. This is consistent with the prediction rate for BI of AT, SN, and PBC found in Sheeran et al.’s meta-analysis, which ranges between 40% and 50% [12]. All three variables, AT, SN, and PBC, have significant predictive effects on BI, with PBC having the highest prediction rate. This finding is in line with Plotnikoff et al.’s research [49]. The prediction rates of AT and PBC on BI are similar and significantly higher than SN, which is consistent with previous studies [15,48]. The TPBT model improved the explanation rate of PA behavior from 34% to 45% compared to the TPB model. The TPBT model explains PA behavior more effectively than other models, including that of Li (41%), who added planning, self-efficacy, and social support to the TPB [45], that of Feng and colleagues (17%), who added planning and self-determined motivation [50], and that of Gomes et al. (11%), who added planning and emotional experience [51]. Additionally, all the model fit indexes of the TPBT model were more appropriate than those of the TPB model. Given the higher explanation rates and better model fit indexes of the TPBT model compared to the TPB model, it is evident that triggers play a crucial role as moderating variables that transform behavioral intention into actual physical activity.

Currently, there is limited research on the influence of triggers on PA behavior in China and other countries. This study discovered that triggers comprehensively act as a moderating variable in the transformation of BI into PA. By triggering PA among college students, they are more likely to respond promptly and increase the likelihood of engaging in PA. This highlights the importance of triggers as a significant factor that affects individual PA. According to the TPB, behavior is not only influenced by intention but also constrained by actual control conditions such as personal ability, opportunity, and resources [48]. The closer the trigger is to motivation and intention, the more likely people are to respond, as stated by the FBM [19]. After incorporating the FBM into the TPB model, the individual’s control over actual conditions such as ability, opportunity, and resources is enhanced, resulting in an improved execution of PA behavior and a higher prediction rate of behavior. The TPBT model enhanced the explanatory power of PA behavior in this study. The newly constructed integrated model effectively narrows the gap between BI and PA behavior, addressing the issue of intention–behavior transformation in the TPB model, and hypothesis 2 is thus supported.

Further investigation is warranted to explore the suitability of the TPBT model for predicting and intervening in Chinese college students’ PA behavior. This study has several limitations that should be noted. Firstly, it is recommended that an experimental intervention be conducted to verify the role of the integrated model. Secondly, the validity and reliability of the recently developed trigger questionnaire require further examination. Thirdly, the use of a retrospective reporting method to measure physical activity behavior may not be accurate. Finally, to enhance the explanatory power of PA behavior, it is necessary to incorporate additional psychological factors into the TPBT model in the future.

## 5. Conclusions

This study attempted to integrate the Theory of Planned Behavior (TPB) and the triggers of Fogg’s behavior model. The study examined the role of triggers in the integrated model and found that they have a moderating effect on the relationship between behavioral intention (BI) and physical activity (PA) behavior. This study also suggests that the explanatory power of the original TPB model for PA behavior can be enhanced by adding triggers into the TPB model, and among the three dimensions of triggers, people are more receptive to facilitator and signal triggers than spark triggers.

## Figures and Tables

**Table 1:** Demographic characteristics of the test samples.

Variable	Classification	*n*
**Sex**	Male	198
	Female	398
**Age**	17	9
	18	152
	19	276
	20	129
	21	30

**Table 2:** Normal distribution test statistics (*n* = 596).

Items	S	K
SN	−0.333	−0.280
AT	−0.255	−1.119
PBC	−0.145	−0.946
BI	−0.338	−0.408
Trigger	−0.070	−0.890
PA Behavior	0.945	−0.172

Note: S = skewness, K = kurtosis.

**Table 3:** Pearson’s correlation matrix and descriptive statistics of each variable (*n* = 596).

Variable	SN	AT	PBC	BI	Trigger	PA Behavior	*M*	*SD*
SN	1						4.25	1.190
AT	0.286 **	1					4.35	1.160
PBC	0.345 **	0.436 **	1				3.87	1.272
BI	0.362 **	0.491 **	0.499 **	1			4.22	1.283
Triggers	−0.022	0.069	0.025	0.015	1		3.54	0.745
PA Behavior	0.291 **	0.317 **	0.295 **	0.548 **	0.262 **	1	27.01	26.190

Note: *M* = mean, *SD* = standard deviation, ** *p* < 0.01.

**Table 4:** TPB model fitting index (*n* = 596).

*χ* ^2^	*df*	*p*	*χ*^2^/*df*	*IFI*	*TLI*	*CFI*	*AGFI*	*RMSEA*
297.735	84	0.000	3.544	0.955	0.944	0.955	0.916	0.065

**Table 5:** Fitting index of TPB and triggers model (*n* = 596).

*χ* ^2^	*df*	*p*	*χ* ^2^ */df*	*IFI*	*TLI*	*CFI*	*AGFI*	*RMSEA*
458.759	181	0.000	2.535	0.952	0.944	0.951	0.917	0.051

**Table 6:** Triggers moderating effect test.

Model		Unstandardized Coefficients		Standardized Coefficients	*t*	*p*	$R^{2}$	*F*	*p*
		B	SE	*β*					
PA behavior	(Constant)	27.007	0.856		31.545	0.000	0.365	170.624	0.000
	BI	11.119	0.668	0.545	16.648	0.000			
	Triggers	8.929	1.151	0.254	7.760	0.000			
	(Constant)	26.936	0.835		32.260	0.000	0.397	130.140	0.000
	BI	10.128	0.675	0.496	15.012	0.000			
	Triggers	9.792	1.133	0.278	8.647	0.000			
	BI × Triggers	5.020	0.893	0.187	5.619	0.000			

**Table 7:** Sparks, signals, and facilitators moderating effect test.

Model		Unstandardized Coefficients		Standardized Coefficients	*t*	*p*	$R^{2}$	*F*	*p*
		B	SE	*β*					
PA behavior	(Constant)	27.007	0.884		30.535	0.000	0.323	141.199	0.000
	BI	11.267	0.690	0.552	16.328	0.000			
	Spark	4.156	0.952	0.148	4.367	0.000			
	(Constant)	27.052	0.882		30.673	0.000	0.328	96.277	0.000
	BI	10.972	0.701	0.538	15.644	0.000			
	Spark	4.446	0.958	0.158	4.640	0.000			
	BI × Spark	1.582	0.731	0.075	2.164	0.031			
PA behavior	(Constant)	27.007	0.872		30.967	0.000	0.341	153.670	0.000
	BI	11.191	0.680	0.548	16.451	0.000			
	Signal	5.935	0.982	0.201	6.042	0.000			
	(Constant)	27.003	0.858		31.461	0.000	0.363	112.513	0.000
	BI	10.486	0.688	0.514	15.251	0.000			
	Signal	6.321	0.971	0.214	6.513	0.000			
	BI × Signal	3.359	0.747	0.152	4.498	0.000			
PA behavior	(Constant)	27.007	0.852		31.704	0.000	0.372	175.337	0.000
	BI	10.962	0.665	0.537	16.483	0.000			
	Facilitator	8.300	1.015	0.266	8.174	0.000			
	(Constant)	26.785	0.831		32.228	0.000	0.404	133.852	0.000
	BI	10.051	0.668	0.492	15.054	0.000			
	Facilitator	8.677	0.992	0.278	8.748	0.000			
	BI × Facilitator	4.813	0.846	0.186	5.687	0.000			

## Data Availability

The data are not publicly available due to privacy reasons.
